# Visualization of lithium-ion transport and phase evolution within and between manganese oxide nanorods

**DOI:** 10.1038/ncomms15400

**Published:** 2017-05-24

**Authors:** Feng Xu, Lijun Wu, Qingping Meng, Merzuk Kaltak, Jianping Huang, Jessica L. Durham, Marivi Fernandez-Serra, Litao Sun, Amy C. Marschilok, Esther S. Takeuchi, Kenneth J. Takeuchi, Mark S. Hybertsen, Yimei Zhu

**Affiliations:** 1 SEU-FEI Nano-Pico Center, Key Laboratory of MEMS of the Ministry of Education, Southeast University, Nanjing 210096, China; 2 Condensed Matter Physics & Materials Science Department, Brookhaven National Laboratory, Upton, New York 11973, USA; 3 Department of Physics and Astronomy, Stony Brook University, Stony Brook, New York 11794, USA; 4 Department of Chemistry, Stony Brook University, Stony Brook, New York 11794, USA; 5 Institute for Advanced Computational Science, Stony Brook University, Stony Brook, New York 11794, USA; 6 Department of Materials Science and Engineering, Stony Brook University, Stony Brook, New York 11794, USA; 7 Energy Sciences Directorate, Brookhaven National Laboratory, Upton, New York 11973, USA; 8 Center for Functional Nanomaterials, Brookhaven National Laboratory, Upton, New York 11973, USA

## Abstract

Multiple lithium-ion transport pathways and local phase changes upon lithiation in silver hollandite are revealed via *in situ* microscopy including electron diffraction, imaging and spectroscopy, coupled with density functional theory and phase field calculations. We report unexpected inter-nanorod lithium-ion transport, where the reaction fronts and kinetics are maintained within the neighbouring nanorod. Notably, this is the first time-resolved visualization of lithium-ion transport within and between individual nanorods, where the impact of oxygen deficiencies is delineated. Initially, fast lithium-ion transport is observed along the long axis with small net volume change, resulting in two lithiated silver hollandite phases distinguishable by orthorhombic distortion. Subsequently, a slower reaction front is observed, with formation of polyphase lithiated silver hollandite and face-centred-cubic silver metal with substantial volume expansion. These results indicate lithium-ion transport is not confined within a single nanorod and may provide a paradigm shift for one-dimensional tunnelled materials, particularly towards achieving high-rate capability.

## Abstract (web summary)

Hollandite structured materials are potentially useful for battery technologies. Here the authors report the unusual lateral transport of lithium ions between lithiated silver manages oxide nanorods where the reaction fronts and kinetics are maintained within the neighbouring nanorods.

Porous manganese oxides such as α-MnO_2_ hollandites (termed OMS-2, octahedral molecular sieves-2) have gained significant attention as electroactive materials^1^ as their tunnel-based crystallographic structure may provide sufficient structural rigidity to enable repeated ion exchange within their one-dimensional (1D) forms^2^,^3^,^4^,^5^,^6^,^7^,^8^,^9^. Specifically, 1+ or 2+ cations often partially occupy the tunnels resulting in mixed Mn 3+/4+ oxidation states in the MnO_6_ octahedra^10^,^11^. Typically, ions within the tunnels are inert; however, in the case of silver hollandite, the Ag^+^ centres are electrochemically active.

While some hollandite-type materials have been previously studied for battery use^12^,^13^,^14^, preparation of pure silver hollandite at sufficient scale for electrochemical assessment was elusive until enabled by hydrothermal methods in 2007 (ref. 15) and a low-temperature reflux-based synthesis in 2010 (ref. 16). Recently, the ability to synthetically tune Ag_*y*_Mn_8_O_16_ material properties by manipulation of silver content (*y*) was reported, affirming the critical roles of composition and physical property control for lithium-ion battery applications^17^,^18^. Investigation of Ag_*y*_Mn_8_O_16_ (*y*=1.2 or 1.6) indicated nanorod morphology with longitudinal alignment of the tunnels (*c* axis) and the presence of oxygen vacancies near the surface. Furthermore, the sample with greater quantities of oxygen vacancies exhibited a seven-fold increase in discharge capacity in lithium based batteries^19^, where the significant differences in capacity were retained upon extended discharge–charge cycling^17^,^20^. In this prior work, it was hypothesized that the MnO_6_ octahedral distortions and oxygen vacancies facilitated lateral (*a–b* plane) Li^+^ diffusion^19^.

Here, using an *in situ* scanning/transmission electron microscopy (S/TEM) approach developed at Brookhaven National Laboratory^21^, lithiation of Ag_1.6_Mn_8_O_16_ nanorods is observed ‘operando', revealing not only Li^+^ diffusion along an individual nanorod, but also the first direct experimental observation of lateral (*a–b* oriented) transfer of Li^+^ between nanorods. *In situ* measurements show two distinct regimes of lithiation: an initial, β regime formed by fast Li diffusion and a subsequent γ regime formed following the passage of a reaction front (RF) and characterized by substantial volume expansion. With electron energy-loss spectroscopy (EELS), we further characterize these two regimes of lithiation, finding that in the β regime *x*∼1 Li^+^ while in the γ regime, *x*>6 in Li_*x*_Ag_1.6_Mn_8_O_16_. Local imaging and diffraction clearly show polyphase material in both regimes, including nanoscale fcc Ag^0^ particle formation in the γ regime. Lateral transfer of Li^+^ between Li_*x*_Ag_1.6_Mn_8_O_16_ nanorods in the *a–b* direction is observed. Atomic scale calculations, based on density functional theory (DFT), are used to demonstrate crystal phases with Li concentrated in local planes that disrupt the tunnel walls in the *x*∼1 regime and the enthalpic driving force for the expulsion of Ag in the *x*>6 regime. Coarse-grained thermodynamic simulations capture the formation of polyphase material in the β regime and the evolution of the RF that separates the γ regime.

## Results

### *In situ* visualization of lithium-ion transport pathways

**Figure 1:**
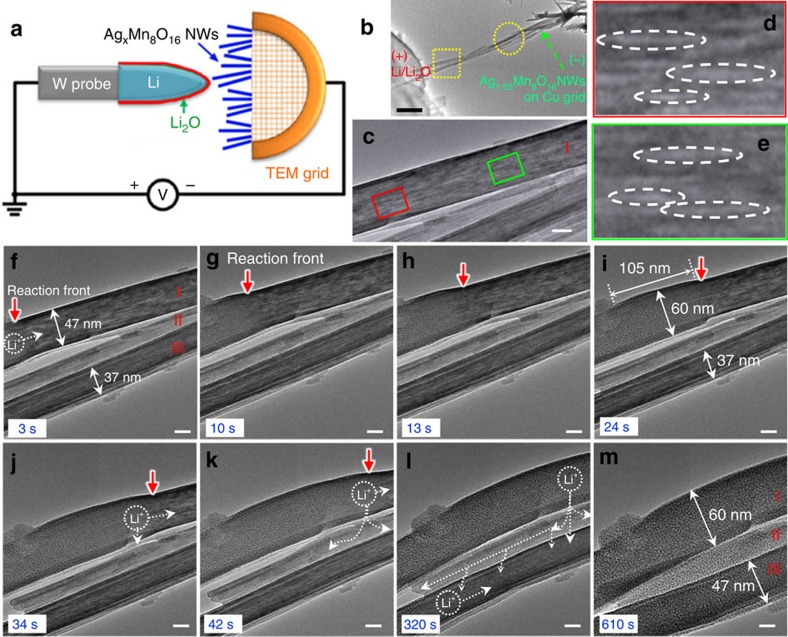
*In situ* TEM observations of the lithiation process in Ag_1.63_Mn_8_O_16_ nanorods. (**a**) Schematic illustration of the experiment setup. Electrochemical lithiation was initiated by applying a constant potential of −1.0 V to the nanorods with respect to the Li counter electrode. (**b**) The panoramic image of the region with Li/Li_2_O on the left. Scale bar, 500 nm. (**c**–**m**) Snapshots of the lithiation process from video, showing the three types of Li-transport pathways for three nanorods (I-III) in the boxed area in **b**. Scale bars, 20 nm. (**c**) Morphology upon applying potential. (**d**,**e**) Magnified images from the red and green boxed area in **c**, showing the early stage of lithiation with needle-like domains. (**f**–**i**) Propagation of the RF (marked by red arrows) with considerable volume expansion of the fully lithiated region. (**j**,**k**) RF in nanorod II propagates through lateral contacts between nanorods I and II. (**l**,**m**) RF in nanorod III propagates through lateral contacts between nanorods II and III, as well as longitudinally from left. Dashed lines and arrows represent the Li diffusion pathways.

Silver hollandite was synthesized and characterized as summarized in Methods. The conceptual schematic for *in situ* lithiation is in Fig. 1a. The real-time morphological evolution observed in the dotted square region in Fig. 1b was sampled by a series of still images shown in Fig. 1c–m (also see Video Clip V1 in the Supplementary Materials). As shown in Fig. 1b, two distinguishable nanorods were contacted directly, and the sample contained an additional nanorod that contacted the other two, but not the Li tip. Thus, we were able to observe two distinct lithium transport pathways.

First, lithium transport in nanorod I propagated along the longitudinal direction starting from the point of contact with the lithium tip. Upon applying the potential, we observed changes in the interior contrast of nanorod I where many needle-like regions became visible, Fig. 1c–e. After 3 s, a distinct lithiation RF appeared, specifically characterized by substantial cross-sectional expansion, and propagated longitudinally, Fig. 1f–k, while the region behind it underwent a 27.6% radial expansion (47–60 nm). The RF propagated ∼105 nm within 21 s, corresponding to a speed of ∼5 nm s^−1^. For the region of nanorod I behind the RF, the image contrast indicated a change from the morphology observed in Fig. 1d,e. Numerous tiny crystallites appeared coincident with expansion of nanorod I, while nanorods II and III retained their initial morphology. This observation confirms that the observed morphological changes were due to electrochemical lithiation rather than beam-induced phase decomposition^22^. Repeated careful experiments suggested that the high-intensity electron beam did not yield a similar structure change of our samples after a prolonged irradiation of 10 min, Supplementary Fig. 1.

After ∼24 s, the RF from nanorod I reached nanorod II where a second pathway for lithium transport was observed. The expansion of nanorod I created lateral contact points between the nanorods, Fig. 1i–m. At 34 s, the RF has crossed the boundary between the two rods and formed a reacted area centred at the top part of nanorod II, as indicated by the small white-vertical arrow (Fig. 1j). During this 10 s, the reaction has extended ∼10 nm deep into nanorod II and ∼48 nm along its *c* axis. The RF reached the bottom edge of the nanorod II at 42 s (Fig. 1k). It also continued to propagate along the *c* axis of nanorod II. Thus, lithiation proceeded in both directions, away from the point of contact with nanorod I. In particular, although the initial source of Li^+^ was different (lateral transfer), the resulting radial expansion of nanorod II was essentially the same as that observed in nanorod I. Furthermore, analysis of the RF progress in nanorod II indicated an estimated velocity of 1.9 nm s^−1^ across the diameter of the rod and a velocity of ∼4.4 nm s^−1^ longitudinally. While these values were somewhat less certain due to the altered focus conditions of nanorod II, they clearly show an asymmetry for RF motion in the *a–b* direction compared to along the *c* axis. Also, the rate along the *c* axis is similar that observed in nanorod I, suggesting that once established, the inter-rod Li transfer is not rate limiting. This first experimental observation of lateral transport between nanorods was fully reproducible. To further test this observation with another sample, a prelithiated nanorod A was used to laterally contact a pristine nanorod B, Supplementary Fig. 2, and the electrochemical lithiation was easily transferred to the pristine nanorod B, proving an unhindered lateral transport pathway between the nanorods. Nanorod B exhibited a radial expansion of 28.1% similar to 27.6% of nanorod I, Fig. 1. The observation of facile, local transfer of Li between nanorods in a localized contact region clearly indicated *a–b* plane diffusion of Li within the nanorods, as hypothesized previously^19^.

Starting at ∼42 s, the lateral and longitudinal lithium transport pathways were observed simultaneously for nanorod III, Fig. 1k,l. Nanorod III was laterally lithiated by the lithiated nanorod II. The expansion of nanorod II also repositioned nanorod III so as to establish contact with the Li electrode. Thus lithiation also propagated along the *c* axis of the nanorod III, similar to nanorod I. The final state of nanorod III also showed expansion, from 37 to 47 nm in diameter, ∼27%. This value is consistent with the expansion of nanorods I and B, Supplementary Fig. 2, indicating similar degrees of lithiation. Figure 1m shows the three fully lithiated nanorods after an elapsed time of 610 s. Despite the different pathways, they exhibit no apparent differences in morphology and no cracks or fracture formation, in contrast to Si which undergoes a much larger volume expansion upon lithiation^23^.

### Structural evolution as a result of lithiation

**Figure 2:**
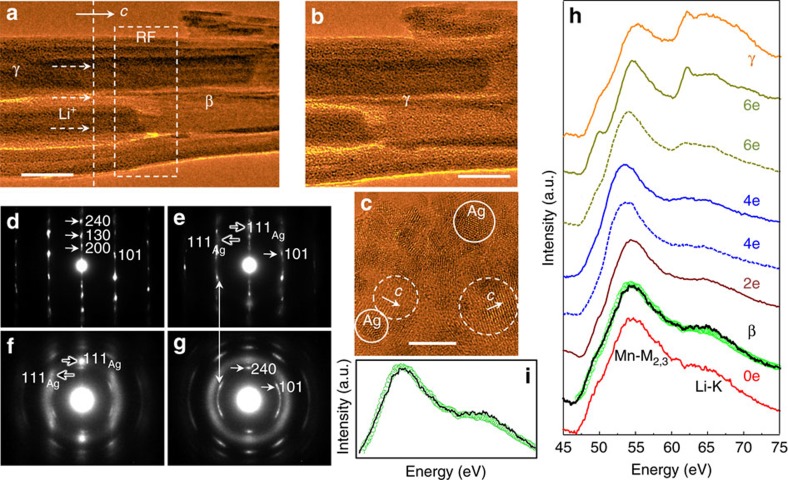
Lithiation process in Ag_1.63_Mn_8_O_16_ nanorods and structural characterization. (**a**) In the fully lithiated area (left, denoted as γ), granular contrast and volume expansion are visible. The RF is marked by the dashed rectangle. Scale bar, 50 nm. (**b**) The same area of the right part of **a**), marked by the vertical dashed line, after RF propagates through the region. Scale bar, 50 nm. (**c**) HRTEM image shows fully lithiated area near the edge of the rods, consisting of small Ag nanoparticles (solid circles) and hollandite grains (dash circles). Scale bar, 5 nm. (**d**–**g**) EDPs show the corresponding structural evolution during lithiation. (**d**) EDP taken from the area marked as β (right in **a**) showing the well-preserved tetragonal structure with crystal-grains aligned along the *c* axis (longitudinal direction) of the nanorods. (**e**–**g**) EDPs from (**e**) RF area, (**f**) lithiated area (**g**, left in (**a**)), and (**g**) after extended lithiation (far left). Ag spots gradually evolve to diffusive rings due to their small size and structural distortion (see (**c**)), indicating increased Ag particle precipitation during lithiation. The Hollandite spots elongate into an arc, indicating that the single crystalline nanorod breaks to small grains with their *c* axis rotating off the longitudinal axis (see the dash circles in **c**). (**h**) EELS of Li-K edge comparing *ex situ* chemical lithiated (0e-6e) and *in situ* TEM-lithiated samples. The spectra are normalized by the Mn-M_2,3_ peak intensity. The Li-K edges from β- and γ-regions are compared with those from chemical lithiated samples (0e-6e), revealing the Li concentration in the β-region is about 0.9e equivalent, while the γ-region is more than 6e equivalent. (**i**) MLLS fitting of the Mn-M_2,3_ and Li-K edge of the β-region with the reference spectra of chemical lithiation 0e and 2e. HRTEM, high-resolution TEM; MLLS, multiple linear least square.

More detailed analysis of the structural evolution during lithiation was obtained from another *in situ* TEM experiment, in which the observed region also had three nanorods with their *c* axis longitudinally aligned, Fig. 2. As before, two distinct regimes were observed: ahead of (right, denoted β) and behind (left, denoted γ) the RF (dashed rectangle). After the RF had propagated, Fig. 2b, the previously unreacted area (right part of Fig. 2a) showed the same morphology and volume expansion as γ. As shown by high-resolution TEM from the fully lithiated region (image in Fig. 2c), the hollandite broke into small grains with the *c* axis off the longitudinal axis of the nanorod, as marked by the dashed circles in Fig. 2c. The spacing of the (001) lattice fringes was slightly increased in comparison with that of pristine hollandite.

The structure of each region was characterized by local area electron diffraction patterns (EDP, Fig. 2d–g). The EDP from the β regime, Fig. 2d, showed well-preserved tetragonal structure with crystal-grains aligned along the *c* axis (longitudinal direction), while the EDP from the RF area, Fig. 2e, showed noticeable changes with diffraction spots beginning to elongate into circular arcs. Furthermore, some diffraction spots attributable to fcc Ag^0^ (indicated by black arrows) developed, although they were quite diffuse. The EDP taken from the γ regime, Fig. 2f, showed both fcc Ag^0^ diffraction spots, and arc-shaped hollandite diffraction spots, indicating breakdown of the *c* axis alignment. Although the Ag^0^ particles had the fcc-based structure, defects and distortion were observed, with corresponding diffraction spot broadening, Fig. 2e–g. Finally, although the EDP taken after the RF had passed showed some further structural evolution, the hollandite component could still be indexed as a tetragonal structure with an increased *a*-lattice parameter (*a*∼1.38 nm), indicating some retention of the parent manganese oxide framework.

To further quantify local lithium concentration, we compared local EELS measurements near the Li K-edge from the different areas observed *in situ* with spectra taken *ex situ* from chemically lithiated reference samples denoted by Faradaic equivalents of lithium ions (Li^+^) and electrons (e^−^) corresponding to 0–6 equivalents per Mn_8_O_16_, Fig. 2h. While the lithiation process for the *in situ* TEM may have been different from the *ex situ* one, nonetheless the chemically lithiated samples were useful to assess the degree of lithiation. Up to 4e^−^, the Li K-edge peak was weak and overlapped with the tail of the Mn-M_2,3_ edge, but clearly the intensity increased with lithiation. In comparison, the integrated peak intensity of Li in the β-region was noticeably higher than the Mn_2,3_ tail from the pristine sample (0e^−^) but lower than that of the 2e^−^ sample. By interpolating the reference spectra for 0e^−^ and 2e^−^ with multiple linear least square fitting, Fig. 2i, we estimated that the β-region corresponded to ∼0.9e^−^. For the 6e^−^ nanorods, a strong lithium peak intensity was typical (the second spectrum from top in Fig. 2h) although the intensity varied from rod to rod indicative of non-uniform lithiation in the *ex situ* samples. The EELS spectra for the γ-region observed *in situ* showed higher integrated intensity than the reference 6e^−^ spectra, thus we concluded that the lithium concentration in the γ-region exceeds 6 Li^+^/Mn_8_O_16_.

DFT, described in Methods, was used to better understand the local atomic structure associated with each phase^24^,^25^,^26^,^27^,^28^,^29^. Previous studies for Li occupancy of pure α-MnO_2_ found orthorhombic distortion, but intact tunnel structures up to *x*=4, and substantial disruption of the MnO_2_ wall structure for larger *x* (ref. 30). The presence of Ag altered this picture significantly. Competing structures for Li_*x*_Ag_*y*_Mn_8_O_16_ across the full stoichiometry range (up to *x*=8 and *y*=2) have been considered. When competing structures in Ag_*y*_Mn_8_O_16_ were compared in a convex hull, a mild enthalpic driving force stabilized several structures with 1≤*y*≤1.75 in which Ag occupied the 2a Wyckoff positions in the tetragonal space group I4/m, specifically high-symmetry sites at the centres of the hollandite tunnels. However, the energy between competing structures was sufficiently small (<10 meV/Ag) to suggest that for *y* in this range, the vacancies in the Ag occupancy within the supercell considered would be essentially randomly distributed, consistent with high-resolution TEM observations that indicated significant variation in Ag occupancy among tunnels^19^.

**Figure 3:**
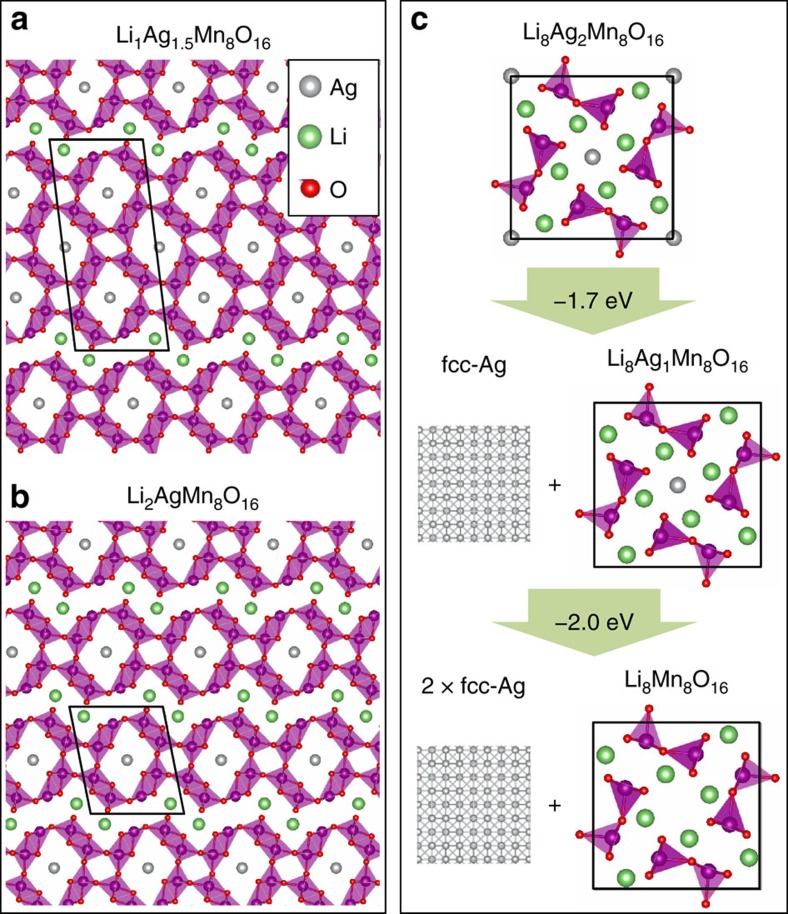
Predicted stable structures for representative Ag and Li concentrations. Once initial lithiation concentration exceeds the local Ag vacancy concentration, there is a driving force for Li to aggregate in sheets and laterally disrupt the tunnel walls. Two examples are shown representing stable phases: (**a**) Li_1_Ag_1.5_Mn_8_O_16_ and (**b**) Li_2_Ag_1_Mn_8_O_16_. They differ primarily in the spacing between the lithiated layers. (**c**) High Li concentration phases, such as Li_8_Ag_2_Mn_8_O_16_ exhibit complete disruption of the tunnel walls and are calculated to phase segregate, expelling Ag to form fcc-Ag.

The initial stable site for Li was a vacancy in the Ag occupancy, but off-centre in the 8 h' Wyckoff position, to better coordinate to oxygen centres. Such stable phases included Li_0.25_Ag_1.5_Mn_8_O_16_, Li_0.5_Ag_1.5_Mn_8_O_16_, and Li_1_Ag_1_Mn_8_O_16_ (Supplementary Fig. 4). For higher Li concentration, the calculations indicated that the Li concentrated in selected planes, as illustrated by two stable phases in Fig. 3a,b. With two Li ions per Mn_8_O_16_ formula unit locally in a single tunnel, the wall structure was disrupted in one direction. All of the structures found up to *x*=2 exhibited rhombohedral distortion, but only a small increase in volume. For large *x*, the wall structure fully opened and the cell cross section area was substantially increased. Overall, the structures for Li_6_Ag_2_, Li_7_Ag_1_ and Li_8_ were nearly the same, with minimal rhombohedral distortion, but substantial volume expansion. However, the calculated energies showed a clear enthalpic driving force towards phase segregation, displacing the larger Ag^+^ ions from the matrix to form Ag metal, Fig. 3c.

A clear picture has emerged for the observed γ regime. It was polyphase, characterized by internal disorder in which the nanorods broke into smaller grains and the emergence of Ag^0^. The DFT results were fully consistent: they showed phase segregation for the *x*≥6 Li^+^ inserted and large lattice expansion along the *a/b* directions. Further, the substantial disruption of wall structure shown by the calculations and the experimentally observed short crystallite length (10 nm scale, Fig. 2c) were both fully consistent with rapid lateral diffusion of lithium within the rods as well as between rods, as observed in Fig. 1j–l.

For the β regime, with an average Li concentration of *x*∼1, two contrast regions were distinguishable in the TEM bright-field image with little change in volume, Fig. 2a. The DFT calculations indicated that stable phases with the right local concentration of Li and an extended, open morphology would facilitate Li diffusion. The observed phase segregation could correspond to one that was Li-rich (such as illustrated in Fig. 3a,b) and one that was Ag-rich/Li-poor (that is, Li_0.25_Ag_1.5_Mn_8_O_16_) where Li at most fills the Ag vacancies. There were two distinct orientations for the extended planes of local Li concentration and the orthorhombic distortion. So, while the difference of in-plane lattice parameters, *b−a=*1.2 Å, was large, the volume or cross-section area change was small (<1%), with the orientation of the orthorhombic distortion of different local phases nucleating randomly. This agreed with the diffraction contrast distinguishing the phases during the *in situ* observations, but no evident expansion in the width of the rod for the β phase.

### Dynamics and phase field model of nanorod lithation

**Figure 4:**
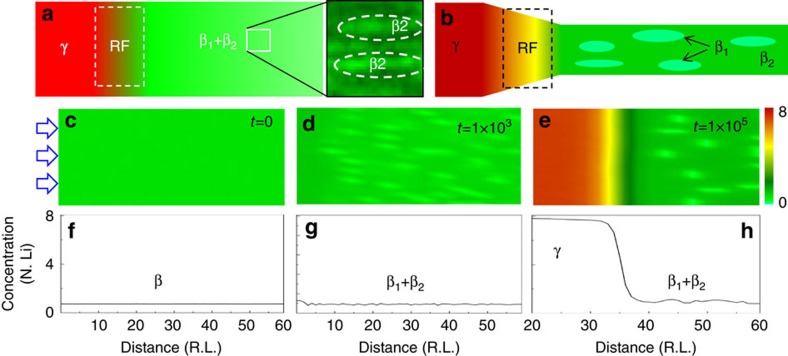
Phase evolution of hollandite nanorods during lithiation. (**a**) An experimental snap shot from the *in situ* TEM study of lithiation of Ag_1.63_Mn_8_O_16_ nanorods (see Fig. 1), showing three distinct regions: the lithiation RF, the area in front of the RF (β_1_+β_2_), and behind (γ). The γ area was measured to have Li concentration greater than 6 per Mn_8_O_16_ unit cell, while the β_1_+β_2_-region has ∼1 Li. (**b**) Schematic representations of the co-existing phases in **a**. (**c**–**e**) Snap shot from two-dimensional phase-field simulation illustrating dynamical evolution of the microstructure (β_1_+β_2_ phase and the motion of the RF) for three instants of time, *t*=0, 10^3^ and 10^5^. The electrochemical boundary condition is applied on the left-hand side, representing the source of Li ions, marked by the arrows. The top row (**c**,**d**) shows two-dimensional maps with the colour legend indicating normalized Li concentration and the bottom row (**f**–**h**) shows one-dimensional Li-concentration (number of Li per unit cell: N. Li) as a function of distance (rescale length in phase-field simulation: R.L.).

We have seen that lithiation occurred in two steps separated by a RF, Fig. 4a,b. During the early stage of lithiation, neither the initial diffusion of Li^+^ to form the β regime nor the initial phase segregation leading to nucleation and growth of the β_1_+β_2_ phases, could be resolved. This suggests a high diffusion rate of Li^+^ into the region up to a concentration of about *x*∼1. Using a lower bound diffusion coefficient *D*=1 × 10^−13^ m^2^ s^−1^ based on the Einstein–Smoluchowski relation, *D*=*L*^2^/2*t* (ref. 31), diffusion along the 500 nm extent of the nanorod inside the highlighted box in Fig. 1b would take ∼1 s. The dynamic time scale that we directly observed was the motion of the RF, which moved ∼105 nm from the snapshot in Fig. 1f to the snapshot in Fig. 1i in a period of 21 s. Interpreting this as diffusive motion implied *D*∼2.6 × 10^−16^ m^2^ s^−1^.

Lithium diffusion coefficients were determined for composite silver hollandite electrodes in bulk-lithium-based cells for comparison with the *in situ* results. Galvanostatic intermittent titration technique (GITT) type testing under 40 or 100 mA g^−1^ currents was utilized, see Supplementary Fig. 5. The diffusion coefficients determined from these bulk measurements ranged from 7 × 10^−11^ initially to 1 × 10^−13^ m^2^ s^−1^ after reduction by 1 e^−^, with evidence of lower polarization initially (<0.5 e^−^) and higher polarization at higher levels of reduction (>0.5 e^−^) consistent with the multiple phase model described below. Electrochemical impedance spectra (EIS) collected at several states of discharge were also used, Supplementary Fig. 5. The diffusion coefficients from EIS ranged from 8 × 10^−11^ initially to 1 × 10^−15^ m^2^ s^−1^ after reduction by two electron equivalents. Although the driving force for lithiation was different for the *in situ* and bulk tests, since the former used a ‘constant potential' of −1.0 V versus Li (for different constant potential and multicycle *in situ* experiment, see Supplementary Fig. 3) while the latter used ‘constant current' of 9.1 mA g^−1^ during the discharge steps. For the bulk system, the apparent lithium-ion diffusion coefficients were determined using three experimental methods. The EIS method perturbed the system the least, as the measurement was done at open circuit voltage after 22 h of rest. In contrast, the GITT methods used higher current to perturb the system and shorter relaxation times. An examination of the resulting diffusion coefficients between ∼0 and 1 electron equivalents of discharge indicates that the effective diffusion coefficient determined under EIS conditions was slightly higher than that for the GITT measurements. Notably, for the EIS measurements which were determine beyond two electron equivalents, the effective diffusion coefficient continued to decrease with discharge. Therefore the value determined from the *in situ* results of 2.6 × 10^−16^ m^2^ s^−1^ is reasonable and consistent with the bulk data given the high driving force and advanced state of lithiation as part of the *in situ* measurement. Thus, the bulk tests affirmed that Li-transport timescales were concentration dependent up to a moderate state of discharge and were in general agreement with the values determined from the *in situ* experiment.

To provide more insight into the observed dynamics, we employed a phase field model in which the driven dynamics of the Li concentration inside the material were determined from the Cahn–Hilliard equation, an internal free energy functional, and boundary conditions for the electrochemical interface derived from the Butler-Volmer equation^32^,^33^. For modelling purposes, we assumed the material had a stable phase for Li concentrations of *x*=0, 1 and 8. The multiphase γ-region was treated as a single phase without regard to the internal phase segregation. We started with the initial influx of Li which had reached an average concentration *x*=0.7 and simulated the evolution of the system on the time scale characterized by the RF movement. Model parameters, carefully chosen to qualitatively capture the regime observed in the experiments are listed in Supplementary Table 1.

Starting from an initial state of uniform Li concentration *x*=0.7 (Fig. 4c), subsequent lithiation led to phase separation with co-existence of Ag-rich/Li-free (*x*∼0,β_1_) and Li-poor (*x*∼1,β_2_) phases (Fig. 4d). As lithiation continued, the high Li concentration (*x*∼8, γ-phase) nucleated and grew from the electrolyte/electrode interface. Then, the RF moved down the nanorod, expanding the volume of the γ-phase. At the same time the coarsening of β_1_+β_2_ continued slowly. Our simulations indicated that the details of the microstructure evolution were sensitive to the coefficients in the free energy function. The evolution of β_1_ and β_2_ depended on the energy barrier between the two phases. Other physical features, such as evolution of the RF width and the growth rate of the γ phase, were also analysed. Supplementary Materials contain additional details of parametric dependence (Supplementary Figs 6–9 and simulation Video Clips V2). The simulations presented in Fig. 4 exhibit the balance between the time scale associated to the RF motion and the β_1_-phase coarsening, consistent with the experimental observations, Fig. 1.

## Discussion

We have conducted a systematic study of the discharge process in silver hollandite, including detailed elucidation of the phase evolution upon intra-rod and inter-rod lithiation. Using *in situ* electron diffraction, imaging, and spectroscopy, supported by comparison to chemically lithiated *ex situ* reference samples, we identified two regimes of intra-rod lithiation and multiple lithium-ion transport pathways including lateral inter-rod lithiation. Dynamics of the lithiation process were correlated at the local and bulk levels. DFT and phase field computations validated the observed phase evolution.

In less than one second, Li^+^ diffused 100 nm into the nanorod resulting in an average composition of Li_*x*_Ag_1.63_Mn_8_O_16_ with *x*∼1, but with a morphology that indicated co-existence of two phases. In∼100 s, we observed the steady advance of a RF along the nanorod. Behind the RF, the nanorod diameter expanded ∼30%. Based on electron energy-loss spectroscopy, the area behind the RF corresponded to *x*>6 on average, but imaging and electron diffraction clearly showed polycrystalline material with lithiated hollandite and fcc-Ag^0^ nanoscale regions.

Notably, in both phases, all data pointed to a rapid diffusion of lateral Li^+^ transport across the *a–b* planes, perpendicular to the nanorod axis, despite the *c* axis orientation of the inherent tunnels in the hollandite crystal structure. Furthermore, the same high level of lithiation (*x*>6) was observed in both nanorods due to lateral lithium transfer between rods. Atomic scale calculations based on DFT suggested structural motifs, in the *x*∼1 and *x*>6 regimes consistent with all the observations, including rapid internal Li diffusion in the *a–b* planes. The observed diffusivity values determined at the nanoscale via TEM were consistent with those measured in bulk via GITT and EIS. Thermodynamic phase field simulation results illustrated the phase evolution processes of the microstructure, consistent with TEM observations of the microstructure changes and progression of the RF.

Understanding transport limitations and available diffusion pathways is critical for several classes of 1D materials, including materials structurally^34^ and compositionally^35^ related to the silver hollandite material, and has been an active area of experimental and theoretical study for 1D diffusion in LiFePO_4_ (ref. 36). For materials limited to 1D diffusion pathways, the presence of point defects or dopants blocking the tunnels can have significant impact on diffusion, where very high diffusivity at the nanoscale may not be sustained in large crystals^37^. The work presented here provides an important new context for consideration of kinetic limitations for lithium-ion transport in 1D materials. In some 1D materials, such as the silver hollandite material studied here, inclusion of 2D (*a–b*) planar diffusion is necessary to adequately describe lithium-ion transport. Thus, this work may motivate research to revisit transport in other 1D materials using complementary bulk and nanoscale techniques.

## Methods

### Synthesis and characterization

The synthesis of silver hollandite, Ag_*y*_Mn_8_O_16_·nH_2_O, was performed using an ambient pressure reflux reaction approach as previously described^16^,^17^. Samples were heat treated at 300°C under air prior to microscopic analysis. X-ray powder diffraction patterns were collected on a Rigaku SmartLab and indexed to Ag_1.8_Mn_8_O_16_ (JCPDS no. 87-087)^38^. Crystallite sizes were determined by applying the Scherrer equation to the (211) peak after LaB_6_ correction. Inductively coupled plasma-optical emission spectroscopy (Inductively coupled plasma-optical emission spectroscopy ) was employed on a ThermoScientific iCap 6,000. Thermogravimetric analysis (TGA) was collected on a TA Instruments SDT Q600, where water content was estimated based on weight loss to 360 °C (refs 7, 15). On the basis of results of X-ray powder diffraction, ICP-OES and TGA, the composition was assigned as Ag_1.63_Mn_8_O_15.7_·0.84H_2_O. In addition, a series of chemically lithiated materials were prepared using LiBH_4_ as a lithiating reagent and studied as reference samples for *ex situ* TEM.

### *In situ* configuration

The *in situ* electrochemical cell for directly observing the all-solid nano-LIB was constructed inside a TEM by adapting a commercially available biasing system, Fig. 1a. The hollandite Ag_y_Mn_8_O_16_ nanowires as the cathode were glued to the half copper grid with conductive epoxy. A lithium metal counter electrode was attached to a sharp tungsten tip that was associated to a piezo-driven biasing-probe built into the sample stage (Nanofactory Instruments AB). After the Li_2_O/Li electrode contacted the nanorods, electrochemical lithiation was initiated by applying a constant potential of −1.0 V to the nanorods with respect to the Li counter electrode. This constant potential was applied throughout the *in situ* lithiation process. The native thin Li_2_O coating formed on the Li tip due to local oxidation acted as a solid-state electrolyte in this configuration^21^.

### Electron microscopy

High-resolution S/TEM (STEM and TEM) imaging, diffraction, and spectroscopy, including chemical and Mn valence mapping, were performed using the double aberration-corrected JEOL-ARM200CF microscope with a cold-field emission gun and operated at 200 kV. The microscope is equipped with JEOL and Gatan HAADF detectors for incoherent HAADF (Z-contrast) imaging, Gatan GIF Quantum ER Energy Filter with dual EELS for spectroscopy. *In situ* electric biasing experiments were carried out using the modified Nanofactory piezo-controlled sample holder.

### Electrochemical assessment

Two electrode electrochemical cells were assembled with lithium metal anodes and cathodes utilizing Ag_1.66_Mn_8_O_16_. Electrochemical testing was measured on a BioLogic model VSP multichannel electrochemical analyser. The galvanostatic intermittent technique (GITT) used two tests: the first with a current density of 40 mA g^−1^ applied to the cell for 90 s followed by a rest time of 2 h, and the second with a current density of 100 mA/g applied to the cell for 180 s followed by a rest time of 10 h. The diffusion coefficient from GITT was derived using equation (1):^39^,^40^,^41^


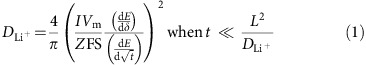


where *L*=finite diffusion length, *I*=applied current, *V*_m_=molar volume of Ag_1.66_Mn_8_O_16_, *F*=Faraday's constant, *S*=electrode surface area, d*E*/d*δ*=slope of the coulometric titration curve and d*E*/d
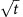
=slope of voltage versus square root of time plot during constant current pulse.

Electrochemical impedance spectroscopy (EIS) was measured during rest steps with a frequency range of 0.1 Hz to 100 kHz where the cell was discharged with a current density of 9.1 mA/g for 2 h followed by a rest time of 22 h. The diffusion coefficients from EIS used equation (2):^42^,^43^,^44^


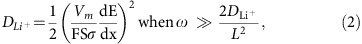


where *ω*=angular frequency, *L*=finite diffusion length, *V*_m_=molar volume of Ag_1.66_Mn_8_O_16_, *F*=Faraday's constant, *S*=geometric surface area of electrode, d*E*/d*x*=slope of the coulometric titration curve and *σ*=Warburg coefficient, obtained from the slope of Re(Z) versus *ω*^−1/2^.

### DFT calculations

DFT total energy calculations and geometry optimization were performed with the VASP package^24^, using the projector augmented wave approach^25^,^26^ and the generalized gradient approximation (GGA) of Perdew, Burke and Ernzerhof (PBE)^27^ for the exchange-correlation density functional. The semi-empirical DFT+*U* method was applied to the Mn 3*d* electrons to approximately account for the strong Coulomb interactions^28^, specifically with the fully anisotropic version, guided by prior results for β-MnO_2_ (ref. 29). Values chosen (*U*=6.2 eV and *J=*1.0 eV) follow the literature^30^. Calculations included spin polarization, with the strong local moment on Mn ions that result assumed to be parallel. Exploration of the impact of a simple, short-range anti-parallel ordering did not substantially alter the atomic structure or the energetics. The fundamental α-MnO_2_ cell contained eight formula units and two independent large tunnels in the tetragonal space group I4/m (No. 87). For the primitive cell (including up to two Ag atoms and/or eight Li atoms), the Brillouin zone was sampled by 2 × 2 × 8 mesh cantered on the origin. The energy cutoff for the Kohn–Sham orbitals was 520 eV. Internal atomic positions and cell parameters were optimized to a tight force criterion, 0.01 eV/A. Crystal structures were visualized using VESTA^45^.

### Phase field calculations

The evolution of the Li concentration inside the material was determined from the Cahn–Hilliard equation and a supporting internal free energy functional:^32^,^33^,^46^


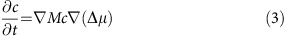



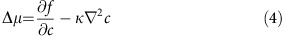


where *M* is the mobility tensor (here taken to be isotropic and constant) and the internal chemical potential Δ*μ* derives from the homogeneous concentration dependent free energy and the Cahn–Hilliard gradient energy coefficient *κ*. The free energy functional is approximated as a piece-wise continuous polynomial. All parameters are rescaled to dimensionless form and the concentration scale is normalized to the range **c**=[0, 1]. The boundary conditions describing the external electrochemical interface kinetics derive from the Butler-Volmer equation:^33^,^46^,^47^


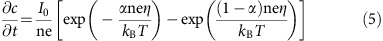


where *α*, is the electron-transfer symmetry factor, ne is the net charge transferred from the solution to the electrode, *k*_B_ is the Boltzmann's constant and *T* is the temperature. The local voltage drop across the interface ΔΦ is included through


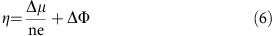


and the exchange current can be written as


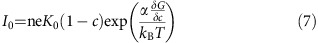


where *K*_0_ contains the details of the interface kinetics, here taken to be a reference constant. The total current integrated over the active facet is controlled by ΔΦ


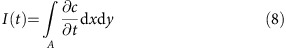


where *A* is the surface area. A constant current boundary condition was implemented by solving equations (5, 6, 7, 8) for the interface voltage drop and then updating the source term for the Cahn–Hilliard equation via equation (5). As a further simplification, once *c*=1 at the interface was reached, it was held fixed. Stable numerical solutions of the Cahn–Hilliard equation were obtained using an implicit finite-difference method and incorporating a small, stochastic variation in the concentration. Details of the parameters chosen and the solutions to the model appear in the Supplementary Materials.

### Data availability

The data that support the findings of this study are available from the corresponding author upon request.

## Additional information

**How to cite this article:** Xu, F. *et al*. Visualization of lithium-ion transport and phase evolution within and between manganese oxide nanorods. *Nat. Commun.* **8,** 15400 doi: 10.1038/ncomms15400 (2017).

**Publisher's note**: Springer Nature remains neutral with regard to jurisdictional claims in published maps and institutional affiliations.

## Supplementary Material

Supplementary Information Supplementary Figures, Supplementary Table 1, Supplementary Notes and Supplementary References

Supplementary Movie 1 clips of microstructure evolution - in-situ experimental observations

Supplementary Movie 2 clips of microstructure evolution - phase-field simulations with different parameters.
