# Supplementary material for: Visualization of lithium-ion transport and phase evolution within and between manganese oxide nanorods
Source: Nat Commun. 2017 May 24;8:15400. doi: 10.1038/ncomms15400 (PMC5458079; doi:10.1038/ncomms15400)
Supplement: Supplementary Information — Supplementary Figures, Supplementary Table 1, Supplementary Notes and Supplementary References [file ncomms15400-s1.pdf]

Supplementary Figures

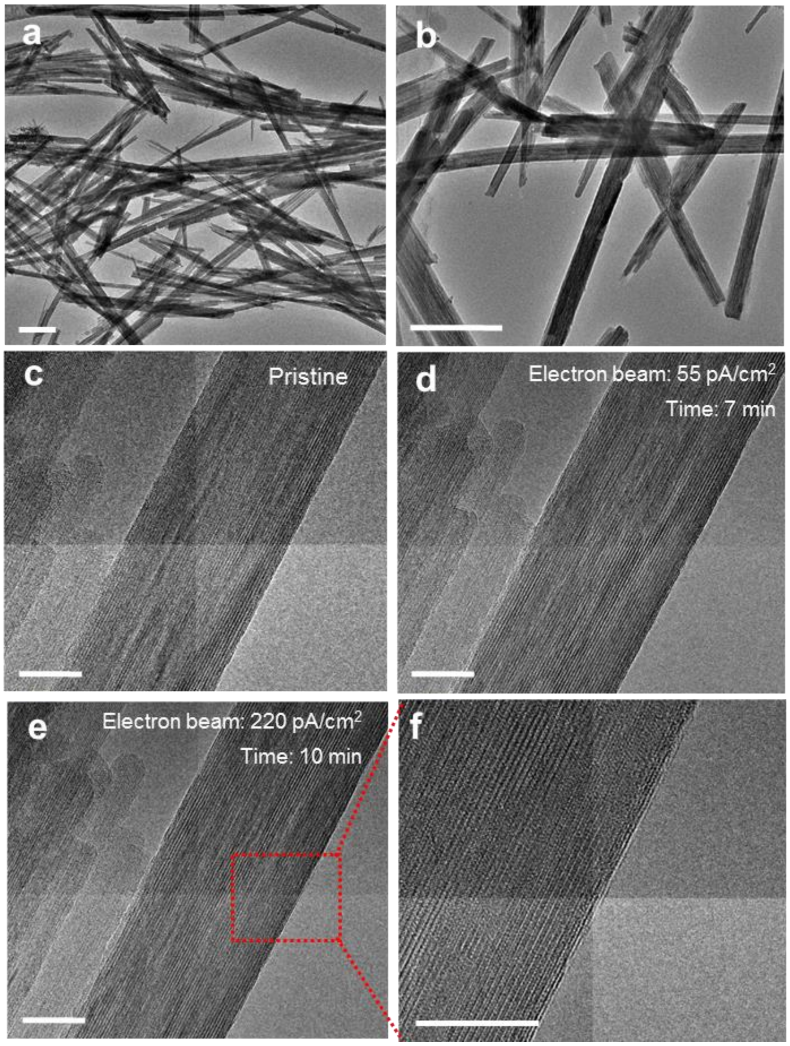

Figure 1. TEM Test for Beam Damage. **(a,b)** TEM images of the pristine $Ag_{1.63}Mn_8O_{16}$ nanorods. Scale bar, 200 nm. **(c-f)** Effects of different electron beam intensities and times on Ag hollandite sample. Scale bar, 10 nm. **(f)** is the enlarged box area in **(e)**. No visible structure damage due to the electron beam was observed. The line contrast in the lattice imaged **(c-f)** are preexisting defects, which can be due to the inhomogeneity of Ag. Such Ag inhomogeneity, as reported previously (*ACS Nano* (9, 8430 (2015)). They may act as the nuclear center for the Li-rich and Ag-rich phase separation.

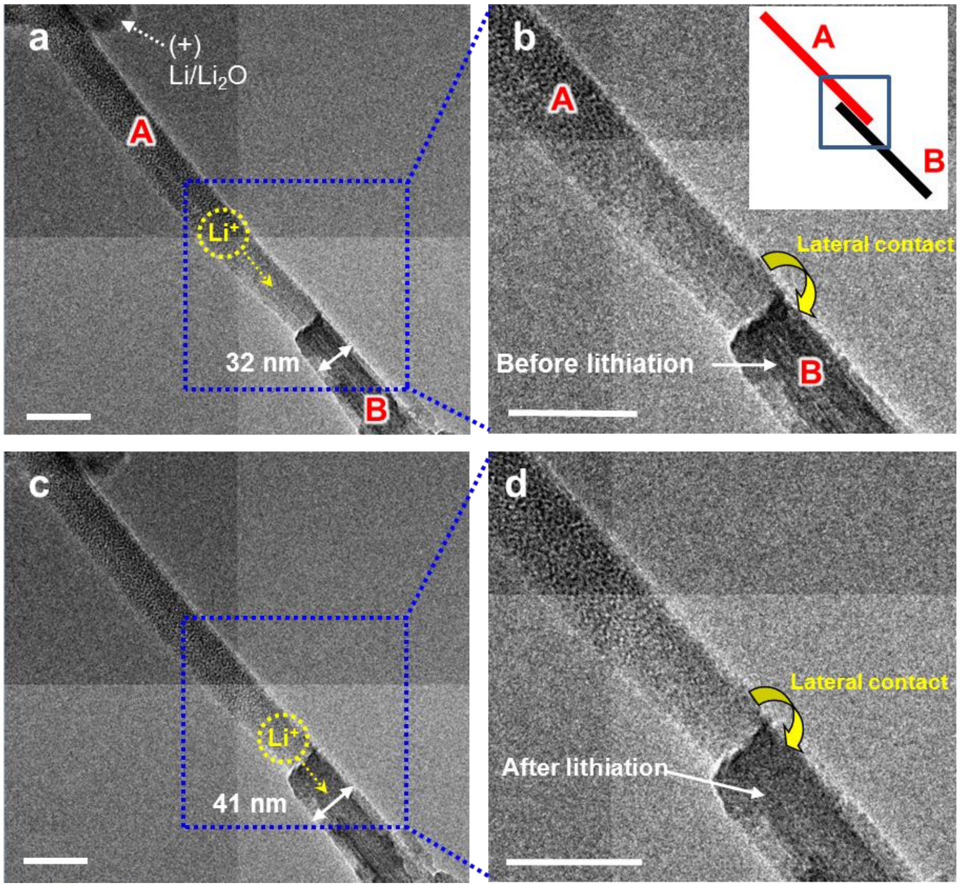

Figure 2. **Additional *in-situ* lithiation observations from TEM.** Two $Ag_{1.63}Mn_8O_{16}$ nanorods were laterally contacted deliberately, and the electrochemical lithiation process was transferred from the lithiated A to pristine nanorod B, proving unhindered lateral lithium transport pathway due to good electrical conductivity between the nanorods. **(a, b)** before lithiation and **(c, d)** after lithiation of the nanorod B. Scale bar, 50 nm. A schematic of laterally contacted nanorods is shown in the inset of **(b)** (see also V2, which is presented at 30X actual speed and the scale bar is 100 nm).

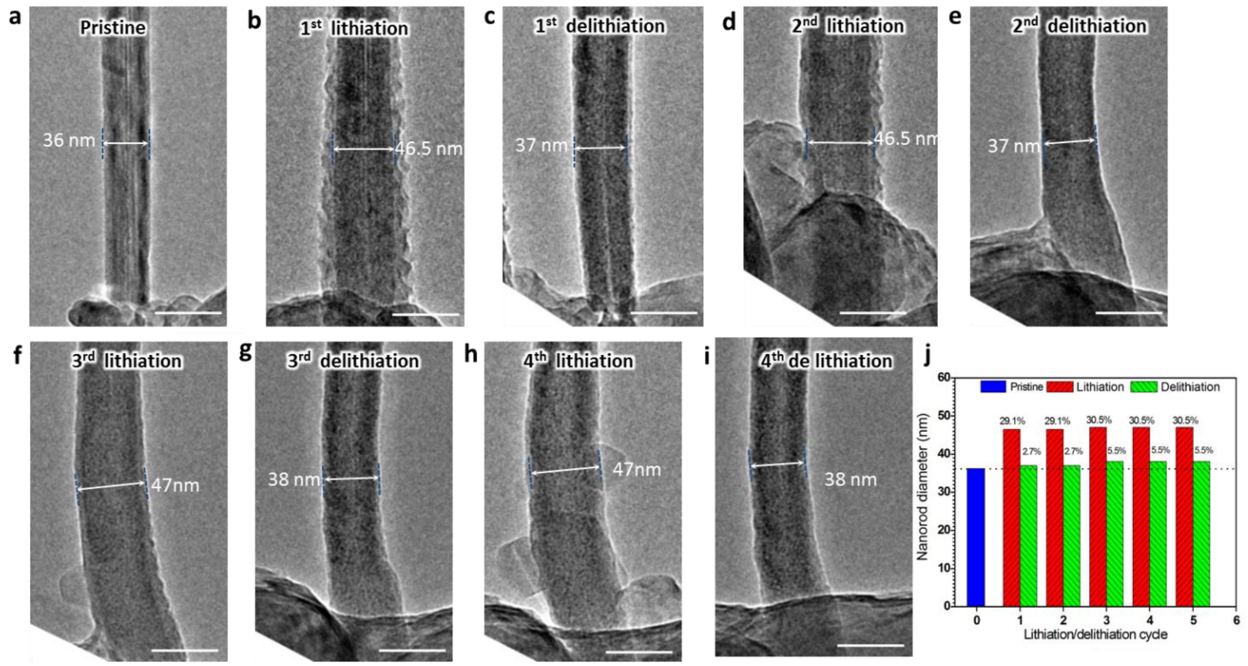

Figure 3. ***In-situ*** **TEM observations of structural and morphology change at the end of multiple lithiation/delithiation cycles of an $Ag_{1.63}Mn_8O_{16}$ nanorod. (a)** A pristine nanorod. **(b-i)** after each individual lithiation/delithiation cycles. Ag nano particles precipitated from the nanorod and volume expansion of the nanorod are clearly visible after each lithiation process. The Ag precipitation is usually irreversible and formation of Li_2O on the surface is also noted after the 1st lithiation. Scale bar, 50 nm. **(j)** Plot of the diameter change of the nanorod during the multicycles. The diameter of the nanorod expands about 29% after the first lithiation. 2.7-5.5% diameter expansion remains at the end of each delithiation process, indicating incomplete extraction of Li-ions during the process, The nanorod was lithiated and delithiated at a constant potential of -2.0 V and +2.0 V. Note the diameters were measured from several fully lithiated (red) and delithiated (green) positions of the nanorod and then averaged for plotting. The percentage of the expansion is reference to the diameter of the pristine nanorod (blue). Careful re- adjustment of the contact between the piezo tip and the nanorod was made during each cycle.

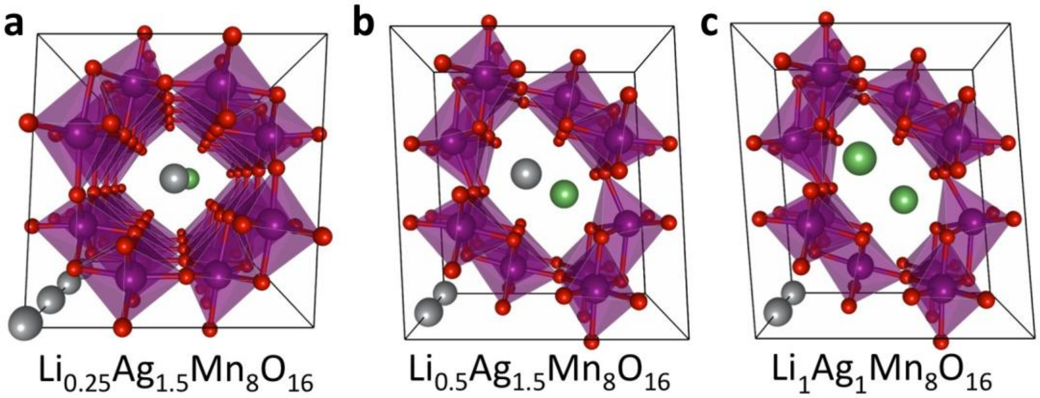

Figure 4. **Stable structures for low Li concentrations predicted from DFT.** At low Li concentration, the tunnel structure is intact and Li occupies "shifted" Ag vacancies (Wyckoff site 8h'). **(a)** $Li_{0.25}Ag_{1.5}Mn_8O_{16}$ has almost perfect tetragonal symmetry. **(b)** $Li_{0.5}Ag_{1.5}Mn_8O_{16}$ shows orthorhombic distortion. **(c)** $LiAgMn_8O_{16}$ Li ions arrange in "zig-zag" manner with significant orthorhombic distortion.

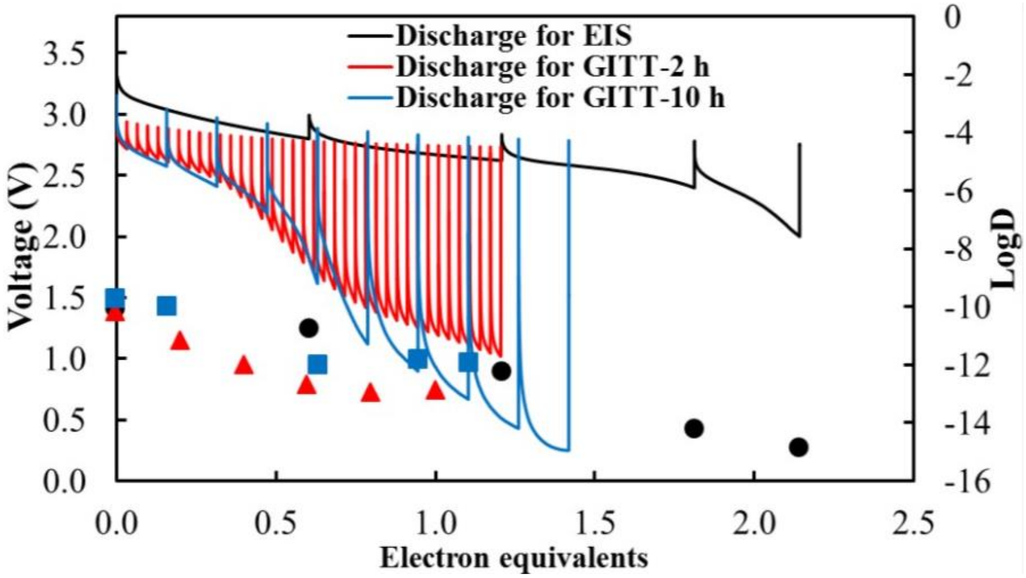

Figure 5. Ex situ measurement of the diffusion coefficient. Measured voltage (left scale) and derived diffusion coefficient (D, right scale, logarithmic) versus electron equivalents of discharge for Li/$Ag_{1.66}Mn_8O_{16}$ cells tested under intermittent galvanostatic discharge using two methods. Galvanostatic intermittent titration test (GITT) was performed with 90 s 40 mA/g discharge and 2 h rest (red line and red triangles) and with 180 s 100 mA/g discharge and 10 h rest (blue line and blue squares). Electrochemical impedance spectroscopy (EIS) was performed under 9 mA/g discharge and 22 h rest (black line and black circles). Analysis to derive D is described in the text.

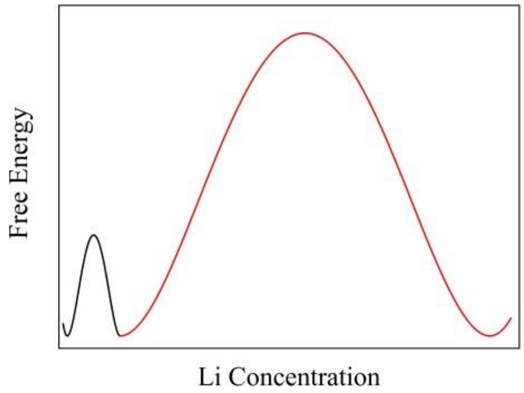

Figure 6. The free energy using piecewise functions of polynomial.

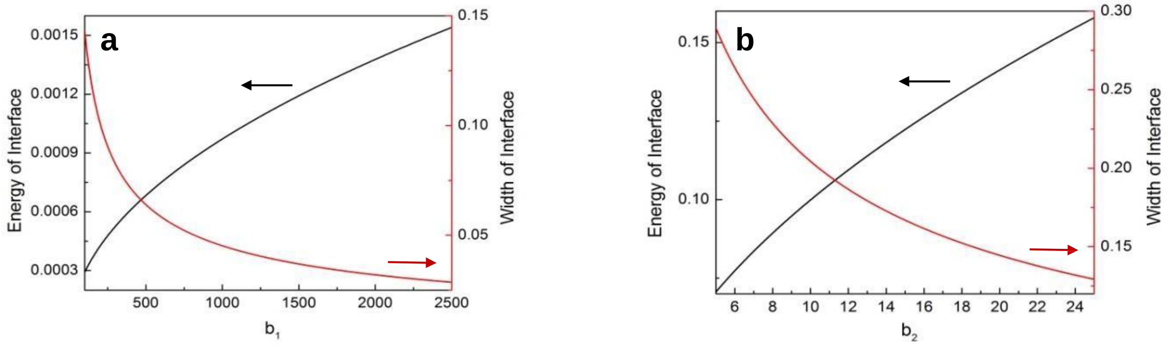

Figure 7. The energy (black line) and width (red line) of interface vs the coefficient parameters of free energy function. (a) b_1 and **(b)** b_2.

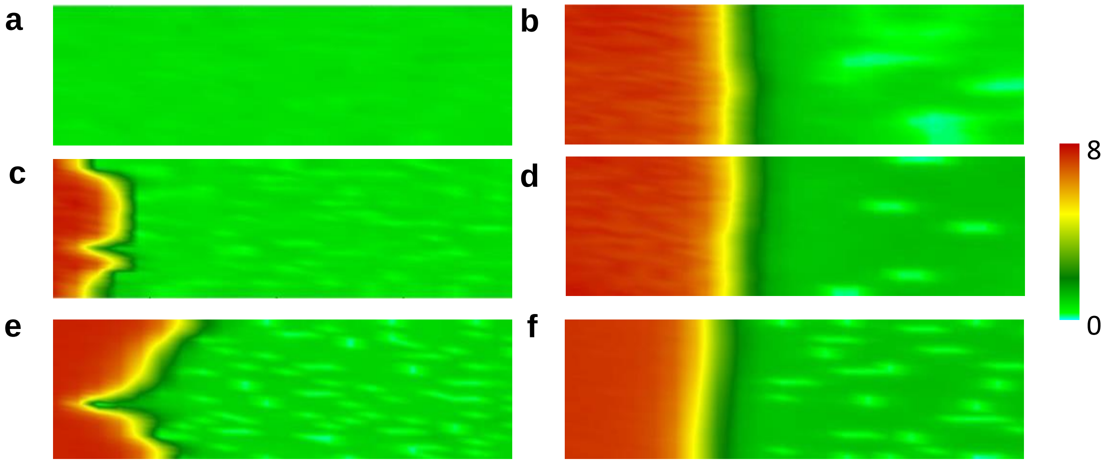

Figure 8. The simulated microstructure evolution using the phase-field model with fixing b_2=100 at two instants of time t=1.1×10^3 (left), and 2.5×10^4 (right) for three different parameters b_1, **(a)** ~ **(b)** 1000; **(c)** ~ **(d)** 5000; and **(e)** ~ **(f)** 8000.

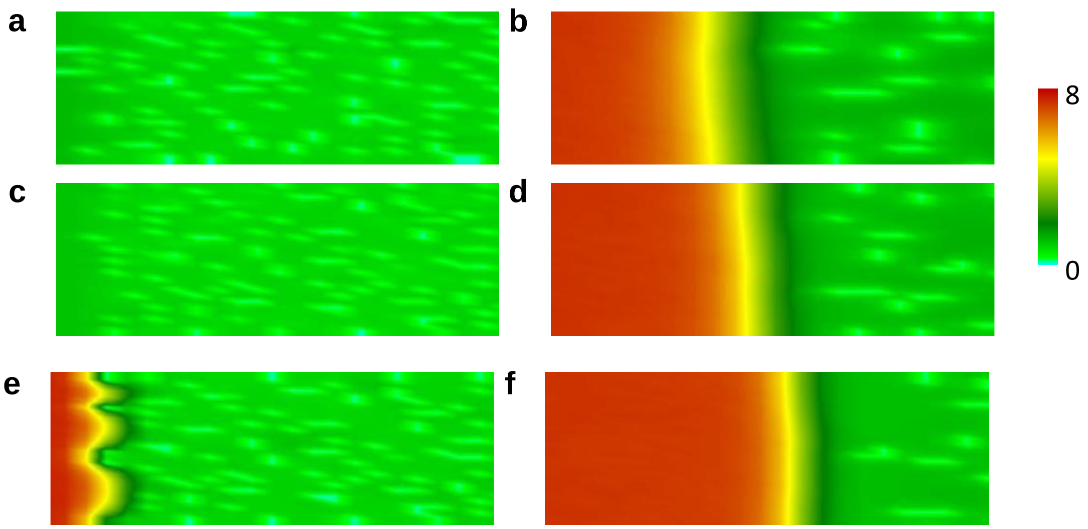

Figure 9. The simulated microstructure evolution with fixing b_1 = 8000 at two times t = 1.1 × 10^3 (left), and 1.0 × 10^5 (right) for three different b_2, **(a) ~ (b)** 30; **(c) ~ (d)** 50; and **(e) ~ (f)** 80.

Supplementary Tables

b_1	b_2	α	κ_1	κ_2	I	M
8000	100	0.5	0.002	0.02	0.01	1

Table 1. Parameters used in the phase field simulation

Supplementary Notes

Additional TEM measurements were made to validate the conclusion of the in-situ TEM experiment. First, the effects of different electron beam intensities and times on Ag hollandite sample were measured, where no visible structure damage due to the electron beam was observed (Supplementary Figure 1). Second, to verify the reproducibility of the observation of lateral transport pathways, an already lithiated nanorod A was used to laterally contact another pristine nanorod B, illustrating the unhindered lateral lithium transport pathway due to good electrical conductivity between the nanorods (Supplementary Figure 2). See Supplementary Videos 1-2.

In our *in-situ* TEM setup we used constant potential for the *in-situ* observations, usually a few electron volts. The structure and morphology of $Ag_XMn8O16$ nanorods do not usually depend on the voltages as the lithiation/delithiation process is mainly dominated by the biasing current which is controlled by the contact resistance between the piezo tip and the individual nanorod. Therefore, high applied voltage does not mean high applied current. Most of our *in-situ* lithiation observations were made at the first discharge (Figure 1, 2 and Supplementary Figure 1, Supplementary Figure 2) although multicycle discharge/charge experiments were also conducted, as shown in Supplementary Figure 3. We note the multicycle results must be carefully interpreted as they often cannot be directly compared with *ex-situ* electrochemical measurement. The main reason is when the voltage is lowered (even before its sign is reversed) or at the start of the delithiation process the contact resistance changes due to the change of the nanorod volume and its surface constituents. As we clearly see in Supplementary Figure 3, the delithiation process at each cycle is incomplete, judging from the residual expansion of the nanorod. During multicycles the lithiation process can be better quantified and compared with *ex-situ* experiment at the first discharge.

The DFT calculations were used to explore the stable structures for $Li_xAg_yMn_8O_{16}$, as described in the main text. We visualize some low Li concentration structures with $x+y \leq 2$ in Supplementary Figure 4.

To obtain bulk measurements of the lithium diffusion coefficienct, two electrode electrochemical cells were assembled with lithium metal anodes and cathodes utilizing $Ag_{1.66}Mn_8O_{16}$. Electrochemical testing was measured on a BioLogic model VSP multichannel electrochemical analyzer. The galvanostatic intermittent technique (GITT) used two tests: the first with a current density of 40 mA/g applied to the cell for 90 s followed by a rest time of 2 h, and the second with a current density of 100 mA/g applied to the cell for 180 s followed by a rest time of 10 h. Electrochemical impedance spectroscopy (EIS) was also performed under 9 mA/g discharge and 22 h rest, as shown in Supplementary Figure 5.

Our *in-situ* experiments and DFT calculations have confirmed that the silver hollandite exists in at least three stable phases, i.e., as-prepared Li-free $Ag_xMn_8O_{16}$ (β_1), Li-poor $LiAg_xMn_8O_{16}$ (β_2), and Li-rich $Li_8Ag_xMn_8O_{16}$ (γ). Thus we hypothesize three phases, corresponding to Li-free $Ag_xMn_8O_{16}$ (β_1), Li-poor $LiAg_xMn_8O_{16}$ (β_2), and Li-rich $Li_8Ag_xMn_8O_{16}$ (γ), in our system. In order to describe the evolution of three phases with insertion of Li ion, a homogeneous free energy with three local minima is required. We use piecewise functions of polynomial to construct the homogeneous free energy. The piecewise functions are written as

$$f(c)=\begin{cases} b_1 c^2 (a_1 - c)^2 & \text{for } c < a_1 \\ b_2 (a_1 - c)^2 (a_2 - c)^2 & \text{for } c \geq a_1 \end{cases} \tag{1}$$

The three local minima are located at $c = 0$, $c = a_1$ and $c = a_2$, respectively. The coefficients b_1 and b_2 determine the maximum (energy barrier) between $c = 0$ and $c = a_1$, and $c = a_1$ and $c = a_2$. The piecewise functions of Supplementary Equation (X1) are continuous and differentiable at the joint point $c = a_1$ between the pieces. Therefore, we can assure the free energy and chemical potential of system is continuous. Supplementary Figure 6 shows an example of the free energy function. In our case, we consider the concentration of three phase equivalent to Li composition $x = 0$, 1, and 8, respectively. We change the coefficients b_1 and b_2 to modulate to heights of energy barriers. Using a piecewise function it is easy to adjust the height of energy barriers.

Eqs. (3) - (8) in the Methods section of the main text describe the Li-ion intercalation, providing a simple paradigm to understand nonequilibrium pattern formation driven by an applied voltage $\Delta\Phi$ or current [1, 2]. Solving Eqs. (3) - (8) in the Methods section of the main text, the change of voltage with average composition and the evolution of Li-ion concentration with time can be obtained at the interface between electrolyte and electrode when the current is known. We employed the explicit finite-difference method to numerically solve Eqs. (3) - (8) in the Methods section of the main text.

The Cahn-Hilliard equation is solved using the implicit finite-difference method to stable numerically solution. In our simulation, all variables are dimensionless, such as rescaled time, length, energy and current unit, $\tilde{t} = kt$, $\tilde{L} = 1$, $\tilde{E} = \frac{E}{\rho k_B T}$. $\tilde{I}_0 = I_0 / K_0$. Figures 4(c) - (e) show simulation results which exhibit the evolution process of the microstructure with increasing Li-ion in Ag Hollandite. In the simulations, we assume that the initial concentration of Li-ion in the entire nanorod is x = 0.7 with uniform distribution because Li-ion with low concentration has a very large diffusion coefficient. The large diffusion coefficient allows the Li-ion with low concentration to fill a 100nm-long nanorod of Hollandite within only 0.1 second, while for the propagation of the γ phase it requires 21 second. The parameters used in the simulations (Fig. 4 (c) - (e)) are shown in Supplementary Table 1.

In our simulation, many parameters affect the calculation outcomes. Singh et al [3] and Bai et al [2] have discussed the effect of applied voltage, the gradient energy coefficient and applied current. Only two phases coexist in their studies while three in our hollandite nanorods. Therefore, the parameters of free energy function, i.e. b_1 and b_2, the gradient energy coefficients are more than that of Singh et al [3] and Bai et al [2]. In the following, we discuss the effect of these parameters. Based on the Cahn and Hilliard theory, the free energy of an interface[4] is

$$\sigma = 2\int_{c_1}^{c_2} [\kappa f(c)]^{\frac{1}{2}} dc \tag{2}$$

and the width of the interface (4) is

$$w = (a_2 - a_1)\left(\frac{\kappa}{f_{\max}}\right)^{\frac{1}{2}} \tag{3}$$

where f_{max} is the maximum value of the homogeneous free energy. Using Eq. (X1), (X2) and (X3), above from the Supplementary Notes, the energy of interface,

$$\sigma = \begin{cases} \dfrac{(\kappa_1 b_1)^{\frac{1}{2}} a_1^3}{3} & \text{for } c < a_1 \\ \dfrac{(\kappa_2 b_2)^{\frac{1}{2}} (1-a_1)^3}{3} & \text{for } c \geq a_1 \end{cases} \tag{4}$$

and the width of interface,

$$w = \begin{cases} \dfrac{4}{a_1}\left(\dfrac{\kappa_1}{b_1}\right)^{\frac{1}{2}} & \text{for } c < a_1 \\ \dfrac{4}{1-a_1}\left(\dfrac{\kappa_2}{b_2}\right)^{\frac{1}{2}} & \text{for } c \geq a_1 \end{cases} \tag{5}$$

Eqs. (X4) and (X5) from the Supplementary Notes above show that the energy and width of the interface monotonously increase with the increase of the Cahn-Hilliard gradient energy coefficient, but with different dependence on the coefficient of free energy function. This agrees with our previous results [5]. Supplementary Figure 7(a) and (b) shows the energy and width of interface vs the coefficient of free energy function. Generally, the width of interface is only a measurable variable from experiment, but the Cahn-Hilliard gradient energy coefficient is difficult to determinate from theoretical calculation or experimental measurement. Therefore, using Eq. (X5) from the Supplementary Notes above, the Cahn-Hilliard gradient energy coefficient can be determined from the experimentally observed width of the interface if the free energy function is known. Because the width of interface only depends on the ratio of the Cahn-Hilliard gradient energy coefficient to the coefficient of free energy function, we only change the coefficient of free energy function.

Supplementary Figures 8 and 9 show the microstructure evolution with different b_1 and b_2, and video clips of the simulations are also provided, Videos 1 - 3. Our simulations show that the phase separation occurs from uniform concentration to heterogeneous $\beta_1+\beta_2$ phase at initiate stage, and then the nucleus of γ phase form at the surface of electrolyte and electrode and grow with increasing Li-ion concentration. Comparing the simulations with various b_1 and b_2 (see Supplementary Figures 8 and 9 and Video 3), we can find that the evolution of β_1 and β_2 phase only depends on b_1, but the growth of γ phase depends on both b_1 and b_2. Supplementary Figure 8 and Video 3 show the case of changing b_1 and fixing b_2; while Supplementary Figure 9 and Video 3 show the case of fixing b_1 and changing b_2. Supplementary Figure 8(a), (c) and (e) shows that the $\beta_1+\beta_2$ phase separation is slow with decreasing b_1, but the coarsening of β_1 phase is faster (see Supplementary Figure 8(b), (d) and (f)). The small b_1 leads to a broad phase boundary of β_1/β_2, so that the phase separation becomes slow due to long diffusion distance. After the phase separation, the low energy barrier between β_1 and β_2 phase lead to the fast coarsening of β_1 phase. Obviously, the microstructure evolution of simulation with large b_1 is consistent with our experimental observation. Our simulation also finds that large b_1 can pin the growth of γ phase (see Supplementary Figure 8 (e)). Numerical analysis using Eq. (X4) from the Supplementary Notes

above indicates that the local chemical potential is a maximum at pinning point. The atomic diffusion is from the region with high chemical potential to the region with low chemical potential. Nevertheless, the concentration of Li-ion at pinning point almost is zero, the large chemical potential may block diffusion of the Li-ions from environment enter the pinning point. The pinning point can be removed by random noise if the b_1 is not sufficiently large. This kind of pinning can lead to a curved morphology rather than a flat interface. The phenomenon is observable from experimental video.

Supplementary Figure 9 shows the evolution of the microstructure with various b_2, and the corresponding video is Video 3. These figures show that large b_2 increases the growth speed of γ phase. Large b_2 can increase the gradient of chemical potential. The ionic flux

$$\mathbf{J} = -Mc\nabla\mu \qquad (6)$$

is proportional to the gradient of chemical potential [1, 3]. Therefore, large b_2 can lead to large the ionic flux, i.e., the faster of the γ phase growth rate.

Based on the above discussions and comparison with experiments, we believe that the microstructure evolution of electrochemical kinetic processes with multi-phases can be well described using free energy function with a series of polynomials. The method can also be applied to other materials.

Video clips of microstructure evolution

V1-2. Video clips of microstructure evolution – in-situ experimental observations.

V3. Video clips of microstructure evolution – phase-field simulations with different parameters.

Supplementary References

1. Bazant, M. Z., Theory of chemical kinetics and charge transfer based on nonequilibrium thermodynamics. *Acc. Chem. Res.* **46**, 1144-1160 (2013).

2. Bai, P., Cogswell, D. A. & Bazant, M. Z. Suppression of phase separation in $LiFePO_4$ nanoparticles during battery discharge. *Nano Lett.* **11**, 4890-4896 (2011).

3. Singh, G. K., Ceder, G. & Bazant, M. Z. Intercalation dynamics in rechargeable battery materials: General theory and phase-transformation waves in $LiFePO_4$. *Electrochim. Acta* **53**, 7599-7613 (2008).

4. Cahn, J. W. & Hilliard, J. E. Free energy of nonuniform system. 1. Interfacial free energy. *J. Chem. Phys.* **28**, 258-267 (1958).

5. Meng, Q. & Zhu, Y. Structural modification of twin boundaries in $YBa_2Cu_3O_{6+\eta}$ oxides: Effects of oxygen concentration and temperature. *Phys. Rev. B* **75**, 174501 (2007).
